# fPADnet: Small and Efficient Convolutional Neural Network for Presentation Attack Detection

**DOI:** 10.3390/s18082532

**Published:** 2018-08-02

**Authors:** Thi Hai Binh Nguyen, Eunsoo Park, Xuenan Cui, Van Huan Nguyen, Hakil Kim

**Affiliations:** 1 Information and Communication Engineering, Inha University, 100 Inha-ro, Michuhol-gu Incheon 22212, South Korea; bichwi@inha.edu (T.H.B.N.); es.park@modulabs.co.kr (E.P.); xncui@inha.ac.kr (X.C.); 2 Faculty of Information Technology, Ton Duc Thang University, Ho Chi Minh City 756636, Vietnam

**Keywords:** liveness detection, fake fingerprints, convolutional neural networks

## Abstract

The rapid growth of fingerprint authentication-based applications makes presentation attack detection, which is the detection of fake fingerprints, become a crucial problem. There have been numerous attempts to deal with this problem; however, the existing algorithms have a significant trade-off between accuracy and computational complexity. This paper proposes a presentation attack detection method using Convolutional Neural Networks (CNN), named fPADnet (fingerprint Presentation Attack Detection network), which consists of Fire and Gram-*K* modules. Fire modules of fPADnet are designed following the structure of the SqueezeNet Fire module. Gram-*K* modules, which are derived from the Gram matrix, are used to extract texture information since texture can provide useful features in distinguishing between real and fake fingerprints. Combining Fire and Gram-*K* modules results in a compact and efficient network for fake fingerprint detection. Experimental results on three public databases, including LivDet 2011, 2013 and 2015, show that fPADnet can achieve an average detection error rate of 2.61%, which is comparable to the state-of-the-art accuracy, while the network size and processing time are significantly reduced.

## 1. Introduction

**Figure 1:**
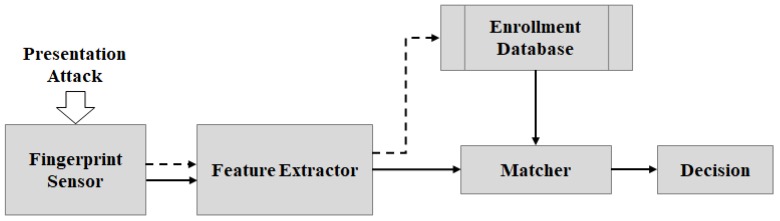
A general fingerprint recognition system. Dashed arrows (⤏) indicate the enrollment process; solid arrows (→) indicate the recognition process.

Authentication systems that use fingerprint recognition are presently evaluated as an authentication method with outstanding growth thanks to the ease of use and economic advantages of low setup costs. These advantages also promote the growth of fingerprint recognition-based applications in mobile devices. A general FPRS (Fingerprint Recognition System) is displayed in Figure 1. The system includes a fingerprint capture device (i.e., a fingerprint sensor), four main modules (namely data acquisition, feature extractor, matcher and decision) and a data storage (enrollment database).

- The data acquisition module is the interface between users and the FPRS. Its function is to scan users’ fingerprints and send them to the extractor.
- The extractor processes the captured fingerprint image to generate a set of features, called the template, which will be used for matching. In some systems, before extracting features, the captured fingerprint image may be evaluated by a quality assessment module. Only images with sufficient quality are passed to the feature extraction module. If the FPRS is performing enrollment, the generated template will be stored in the data storage (enrollment database); otherwise, if the FPRS is performing recognition, this template will be sent to the matcher.
- The matcher computes the similarities between the template delivered by the extractors and the templates stored in the database. These similarity scores are used to recognize the user’s identity.

Since fingerprint-based applications are becoming more common, the security aspect of these systems becomes especially important. The previous studies have proven that fingerprint recognition systems are exposed to several security threats, such as attacking at fingerprint sensors using fake fingerprints (known as presentation attack), attacking the communication channels between modules, attacking the software modules and attacking the data storage [1,2,3]. Among these threats, presentation attacks using fake fingerprints are the most pressing problem for an FPRS because unlike the others, attacking an FPRS system using forged fingerprints does not require any knowledge about the system. There are two methods to fabricate fake fingerprints: cooperative and non-cooperative. In the first method, fake fingerprints are created directly from real fingers; in the second method, fake fingerprints are formed indirectly through lifting a latent sample [4]. Typically, the non-cooperative methods produce the fake fingerprints of lower quality more than the cooperative methods. However, both are considerable threats to fingerprint recognition systems. In 2002, Matsumoto et al. successfully spoofed 11 FPRSs using fake fingers created from gelatin [5]. When the iPhone 5S was released in September 2013, it took only a few days to prove that the TouchID fingerprint sensor of iPhone 5S could be fooled using fake fingers made of latex or wood glue [6]. Therefore, to ensure the security of fingerprint recognition systems, captured fingerprints should be classified as counterfeit or real before any further process.

There are numerous approaches in the literature to determine whether fingerprints are fake. The deep learning-based methods in the recent years have shown a increasing improvement in the detection rate compared to the traditional methods. However, the state-of-the-art algorithms face the problem of high processing time. Moreover, these algorithms require high memory usage and storage. These drawbacks make the existing algorithms unsuitable for being embedded in fingerprint sensors. This paper proposes a network, named fPADnet (fingerprint Presentation Attack Detection network), that can discover fake fingerprints with a high detection rate, in a reasonable time, with low storage usage; thus, it can be integrated with fingerprint sensors. fPADnet uses SqueezeNet [7], which has 100× fewer parameters than that of VGG [8], as the base architecture to minimize the network size and computational time. The use of SqueezeNet helps to reduce the classification time and memory. However, reducing the processing time results in a lower detection rate. How to increase the accuracy while maintaining a low processing time and memory requirement has become a crucial question. The existing studies have proven that texture information plays an essential role in fingerprint liveness detection [9]. CNNs, however, capture the 2D structure of an image. Therefore, the Gram modules are proposed to be integrated with SqueezeNet to remove the spatial information, but keep the textural details of fingerprint images only. Integrating SqueezeNet and the Gram matrix results in a deep neural network that has only 308,554 parameters, which is 2.4 times smaller than that of the original SqueezeNet. Experiments carried out on the three public LivDet Databases (LivDet 2011, 2013, 2015) showed that the average detection error rate of the proposed system was 2.61%, and the processing time was 21 ms on Nvidia GTX 1080. The main contributions of the proposed method are:

- fPADnet is suitable to deploy in real-world fingerprint recognition systems, especially embeddable in fingerprint sensors, thanks to its small size and low processing time. Moreover, the detection accuracy is comparable to the state-of-the-art.
- Fingerprint image sizes are dependent on the sensors, for example, they vary from 252×324 to 1000×1000 pixels in the LivDet 2015 datasets [10]. The existing CNN-based methods require that every image must be resized to the specific input size of the network models. This resizing step is somewhat troublesome and may decrease the detection performance since the natural resolution of the fingerprint images is unintentionally modified. Unlike the existing works, fPADnet can accept images of any sizes as its inputs due to the use of Gram matrices. This characteristic makes fPADnet convenient to use and easy to integrate with any fingerprint sensor.

Before describing the proposed algorithm, this paper provides a literature survey on presentation attack detection in Section 2. The proposed algorithm is presented in Section 3, which includes three subsections. Section 3.1 and Section 3.2 briefly explain SqueezeNet and the Gram matrix, which are the basis to build fPADnet; Section 3.3 describes the architecture of fPADnet. Section 4 reports the experimental results and discussions. Conclusions are discussed in Section 5.

## 2. Literature Review on Presentation Attack Detection

The existing methods can be divided into two categories, namely hardware- and software-based approaches. Hardware-based methods use an additional device to extract the physical characteristics of the human body, such as blood pressure in the fingers, the transformation of skin or skin odor [11,12,13]. These methods are more accurate than those in the software-based category, but they are more expensive due to the cost of extra sensors. Software-based methods detect fake fingerprints by examining the fingerprint images captured by fingerprint sensors. These algorithms have two advantages. First, there are no additional devices required, i.e., no extra cost. Second, it is easy to modify any existing FPRS by assembling a fake fingerprint detection module before the extractor. Due to these advantages, software-based methods have gained more attention from researchers.

Nikam and Agarwal [14,15] proposed a method that combines texture information and wavelet energy features. The texture information was extracted using Local Binary Pattern (LBP) histograms [14] or the gray-level co-occurrence matrix [15]. The wavelet energy features represent ridge and orientation information of a fingerprint image. Both methods in [14,15] used the principal component analysis and the sequential forward feature selection to reduce the dimension of the feature sets. Coli et al. applied the Fourier transform to obtain features for fake fingerprint detection because fake images exhibit less high-frequency characteristics than live images [16]. These authors conducted experiments on their datasets, and detection accuracy rates were from 94% to 97%. Moon et al. [17] observed that the surface of a fake finger is much coarser than that of a live finger. They used a high-resolution camera to capture fingerprint images. A noise removal algorithm was used to calculate the noise residue of a fingerprint image. The standard deviation of the noise residue indicated the texture coarseness; thus, it can be used to distinguish between fake and live fingerprint.

In 2009, the First International Fingerprint Liveness Detection competition (LivDet) was organized. Since then, many publications on fake fingerprint detection have used the databases provided by LivDet for evaluation. Marasco and Sansone [18] used multiple textural features to classify fake and real fingerprints. Their features were derived from several texture analyses, such as the first order statistics, the standard deviation of the residual noise and the ratios between gray-level values. Galbally et al. [19] used ten different quality measures for fake fingerprint detection. They assessed fingerprint quality by measuring ridge strength, ridge continuity and ridge clarity. Quality scores obtained from these measurements were used as features to classify fake or real fingerprints. These authors used the Linear Discriminant Analysis (LDA) as the classifier. Ghiani et al. [20] extracted features for fake fingerprint detection based on a texture analysis of the fingerprint images. Their method relied on the rotation-invariant version of the Local Phase Quantization (LPQ). Gragnaniello et al. [21] showed better performance by combining the Weber Local Descriptor (WLD) and LPQ. Jia et al. [22] stated that the texture of fingerprint images is too complicated to be presented by the LBP. Therefore, to improve the detection rate, they used Multi-Scale LBP (MSLBP) for spoof fingerprint detection. Their results proved that the MSLBP-based method was robust to noise compared to the LBP-based method. A new local descriptor for fingerprint liveness detection was proposed by Gragnaniello et al. [9]. The authors analyzed fingerprint images in both the spatial and frequency domain to extract information on the local amplitude contrast and local behavior of the fingerprint images. A linear-kernel SVM was used to classify between fake and real fingerprints. Binarized Statistical Image Features (BSIFs) were used as the local texture descriptor for fingerprint liveness detection in [23]. BSIF encodes the local fingerprint texture into a feature vector by using a set of filters learned from a small collection of natural images. These continuous attempts resulted in a significant improvement in accuracy. While the error rate of the LivDet 2011 winner was 22.9%, local descriptors-based algorithms can reduce the error rate to 5.7% [9].

The new era of the image classification problem has started with the birth of Deep Convolutional Neural Networks (DCNN). DCNN have rapidly shown their effectiveness in fingerprint liveness detection [24,25,26,27,28,29]. There are two approaches to use convolutional neural networks in fake fingerprint detection. In the first approach, the authors selected an existing network, which was initially trained to detect objects in natural images (such as people, vehicles, animals), and then applied the transfer learning technique to learn the network for fingerprint liveness detection. Several pre-trained networks, such as VGG-19, GoogleNet, CaffeNet and Siamese, were evaluated in [24,25,26]. The experimental results showed a significant improvement in accuracy compared to the non-deep learning-based methods. While the first approach used an entire fingerprint image as the input, the second approach divides a fingerprint image into patches and classifies each patch as fake or live. The final decision is made by the voting strategy, i.e., if the number of fake patches is greater than or equal to that of live patches, the fingerprint is fake. Wang et al. [27], Jang et al. [28] and Park et al. [29] experimented with different network models and different patch sizes. Wang et al. [27] divided a fingerprint image into non-overlapped patches of a size of 32×32 pixels and then used a four-layer CNN to classify each patch. Jang et al. [28] used a network with four convolutional layers and two fully-connected layers, which was inspired by the architecture of the VGG network. The patch size in their work was 16×16 pixels. Park et al. [29] used 11 overlapped patches of a size of 96×96 pixels as the inputs for their proposed network. The patch-based methods mentioned above require fingerprint images to be preprocessed by fingerprint segmentation [27,28,29] and histogram normalization [28].

The average classification error rates of the state-of-the-art CNN-based method on four LivDet databases (LivDet 2009, 2011, 2013, 2015) were 1.63%, 4.53%, 2.33% and 4.49%, respectively [10,25]. This CNN-based algorithm also won first place in the Fingerprint Liveness Detection Competition 2015 thanks to its high performance [10]. Although the existing CNN-based methods have high accuracy, it is difficult to apply them in real-world systems because of their low processing speed and high memory usage. The winner of the Fingerprint Liveness Detection Competition 2015 used the pre-trained VGG model [8] in their work. The amount of time required to classify one fingerprint image was 650 ms on a single-core machine (1.8 GHz, 64-bit, with 4 GB memory). The storage required of this network was over 500 MB. These drawbacks make it difficult to deploy CNN-based algorithms on real-world fingerprint recognition systems, especially embedding in fingerprint sensors and integrating into mobile applications.

## 3. Proposed Network Model

### 3.1. SqueezeNet

**Figure 2:**
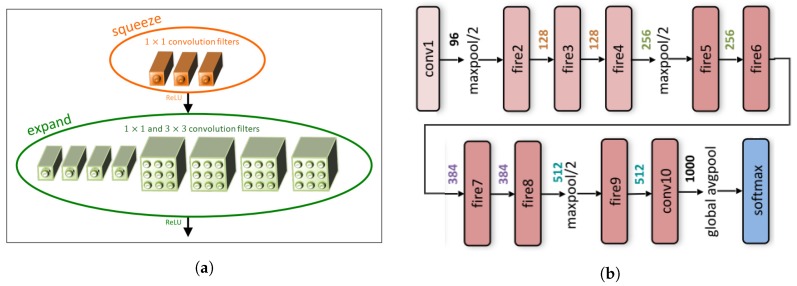
(**a**) Fire module in SqueezeNet; (**b**) SqueezeNet architecture (Figure from [7]).

SqueezeNet is a small CNN architecture that achieves AlexNet-level accuracy on ImageNet with 50× fewer parameters [7]. The fire module, which is the foundation of SqueezeNet, is designed according to three main strategies, which are (1) smaller network by replacing 3×3 filters with 1×1 filters, (2) reduction in the number of inputs for the remaining 3×3 filters and (3) late downsampling in the network so that convolution layers have large activation maps. The Fire module consists of a Squeezelayer, which reduces the number of input channels using a small number of 1×1 convolutions, and an Expandlayer, which increases the number of channels of the Squeeze layer result using 1×1 and 3×3 convolutions. This method is called a bottle-neck structure. The Expand layer of the Fire module also has 1×1 convolution filters to reduce the number of parameters further (Figure 2a). In SqueezeNet, the ratio between the number of filters in squeeze layers and the number of filters in expand layers, named the Squeeze ratio, and the ratio between the number of 1×1 filters and 3×3 filters in the expand layer affect the model size and accuracy. After considering the trade-off between size and efficiency, in the proposed fPADnet, these ratios were set to 0.125 and 0.5, respectively. Fire modules and several pooling layers were stacked to arrive at a small network (Figure 2b).

### 3.2. Gram Matrix

**Figure 3:**
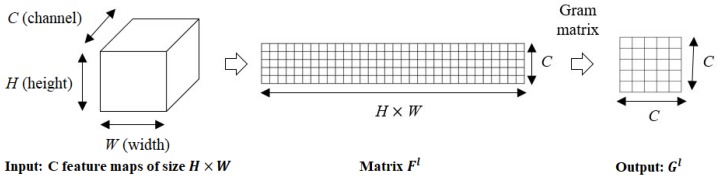
The process of Gram matrix computation.

In 2015, Gatsy et al. [30] explored how the texture of an image could be represented by the correlations between feature maps in a layer of a Convolutional Neural Network (CNN). These feature correlations were computed based on the Gram matrix as follows. The input image is passed through a convolutional neural network; each convolutional layer outputs a set of filtered images called feature maps. Assume that the output of the *l*-th convolutional layer of the CNN has *C* feature maps; each feature map size is H×W pixels. Firstly, *C* feature maps of the *l*-th convolutional layer are transformed into a two-dimensional matrix $F^{l}\in R^{C\times S}$, where S=H×W. The $i^{th}\left(1\leq i\leq C\right)$ row of the matrix $F^{l}$ corresponds to the *i*-th feature maps. The texture information at the *l*-th layer is given by the Gram matrix $G^{l}\in R^{C\times C}$, the entries of which are given in Equation (1).

(1) $$G_{ij}^{l}=<F_{i}^{l},F_{j}^{l}>$$

where <,> denotes the inner product and $F_{i}^{l},F_{j}^{l}$ are the *i*-th and *j*-th rows of the matrix $F^{l}$. Figure 3 describes the process of computing a Gram matrix. The Gram matrix at a specific layer is a summary statistic that discards the spatial information in the feature maps [30]; therefore, a set of Gram matrices from several convolutional layers in the network provides a stationary description of the image texture.

### 3.3. Gram-*K* Module and fPADnet Architecture

**Figure 4:**
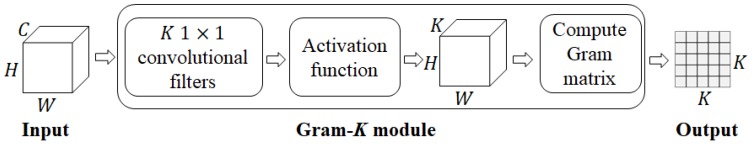
A Gram-*K* module.

The Fire modules of SqueezeNet and Gram matrices form the basis of the fPADnet. However, to make fPADnet independent of input sizes, this paper proposes a new module called Gram-*K*, which is derived from the Gram matrix (*K* represents the output size of a Gram module, for example, *K* is equal to 128 in our experiments). Figure 4 shows the structure of a Gram-*K* module; the activation function used in the experiments is *tanh*. The input of a Gram-*K* module is a set of *C* feature maps, and its output is a Gram matrix of size K×K. The process of computing a Gram matrix is described in Section 3.2. By varying the number of 1×1 convolutional filters, we can control the output size of the Gram module.

**Figure 5:**
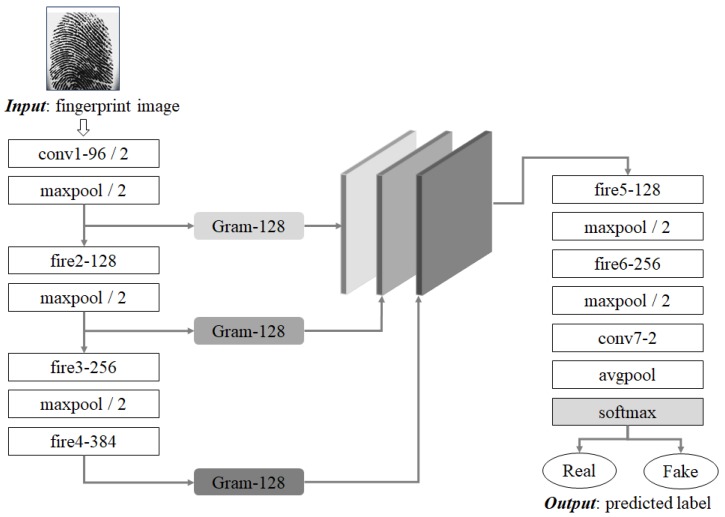
The architecture of fingerprint Presentation Attack Detection network (fPADnet).

Figure 5 shows the structure of the proposed network; the detailed description of fPADnet is presented in Table 1. The network starts with a convolutional layer of 96 filters of size 7×7 and a stride of two. The last convolutional layer of the network (denoted as conv7-2 in Figure 5) uses two 1×1 filters and a stride of one. All max-pooling layers in the proposed network are conducted using 3×3 filters with a stride of two. After the layer conv7-2, a global averaging pooling is applied to map each feature map of the conv7-2 layer to a single value.

Three Gram-128 modules are stacked to the network. Three 128×128 Gram matrices $G_{1},G_{2}$ and $G_{3}$ are obtained at several layers as shown in Figure 5. Those three Gram matrices are concatenated to form a three-dimensional matrix *G*. This 3D matrix *G* is the input of the layer fire5-128 . Note that the output of every convolutional and fire layer is put through ReLU non-linearity. The output of the average pooling layer is sent to the two-way Softmax layer, which produces two probability values for two class labels (fake and real), where a higher value corresponds to a higher probability.

By introducing the Gram-*K* and modifying the SqueezeNet, the total number of parameters of fPADnet is reduced to 308,554, compared to 737,926 parameters in the original SqueezeNet. The network size is merely 1.2 MB if each parameter is stored in 32 bits. This small size allows fPADnet to be embedded into fingerprint sensors and integrated with mobile applications. Thanks to Gram-*K* modules, fingerprint images can be directly put into the network without resizing. Since there is no downscaling, all information from the input image is preserved; therefore, the proposed network is expected to outperform other networks in detecting fake fingerprints. Table 1 provides the details of the proposed network architecture. For each fire module, the number of 1×1 filters in the squeeze layer and the number of 1×1 and 3×3 filters in the expand layer are shown in the column $s_{1\times 1}$, $e_{1\times 1}$ and $e_{3\times 3}$, respectively.

## 4. Experimental Results and Discussions

### 4.1. Datasets

Three public LivDet databases were used, including LivDet 2011 [31], LivDet 2013 [32] and LivDet 2015 [10], to prove the efficiency of the proposed network. LivDet 2011 consists of four datasets captured using four different sensors (Biometrika, Digital Persona, ItalData and Sagem). Gelatine, latex, PlayDoh, silicone, wood glue and EcoFlex were used to made fake fingerprints. All spoof images were collected using the cooperative method. The LivDet 2013 Biometrika and ItalData dataset used the same devices as LivDet 2011. Fake fingerprints in these datasets were made from gelatine, latex, EcoFlex, modasil and wood glue using the non-cooperative method. In addition to Biometrika and ItalData, LivDet 2013 contains two other datasets; one was from a Swipe sensor, and one was from a Crossmatch sensor. However, the images of the Swipe dataset are considerably different from the other sensors; and there was an anomaly when building the LivDet 2013 Crossmatch dataset that caused an improper rate of misclassified live fingerprints [33]. Due to these reasons, the LivDet 2013 Swipe and Crossmatch were excluded from our experiments.

**Figure 6:**
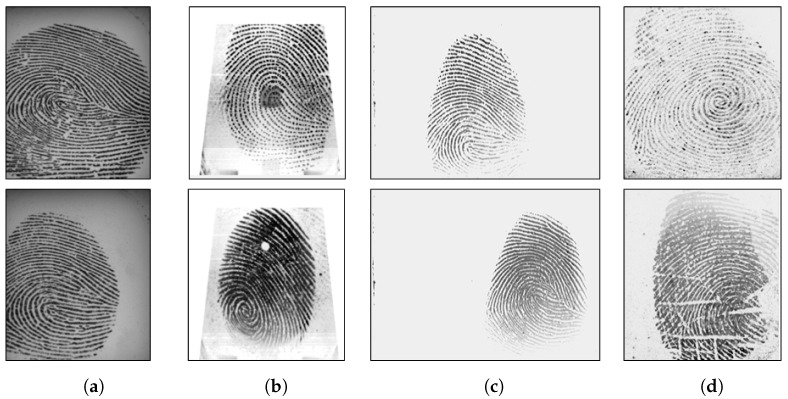
Sample images of LivDet 2011. The top row includes live samples; the bottom row includes fake samples. Samples are from Biometrika (**a**), Digital Persona (**b**), ItalData (**c**) and Sagem (**d**) devices.

**Figure 7:**
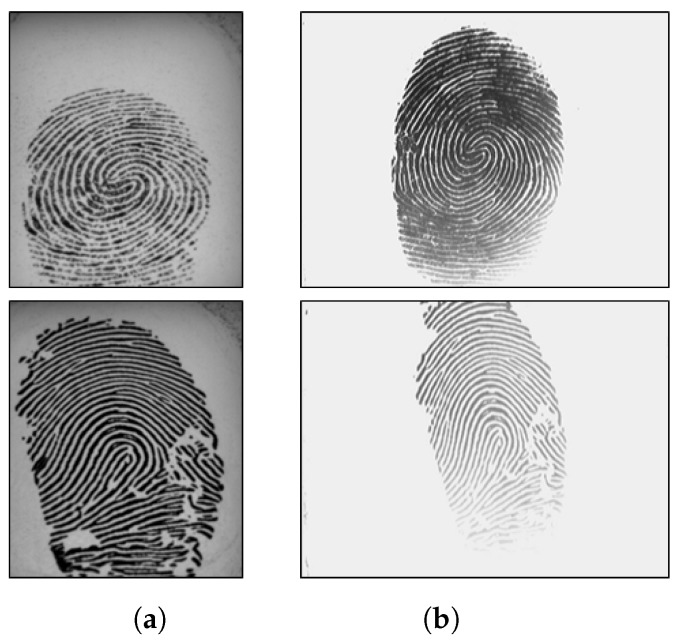
Sample images of LivDet 2013. The top row includes live samples; the bottom row includes fake samples. Samples are from Biometrika (**a**) and ItalData (**b**) devices.

**Figure 8:**
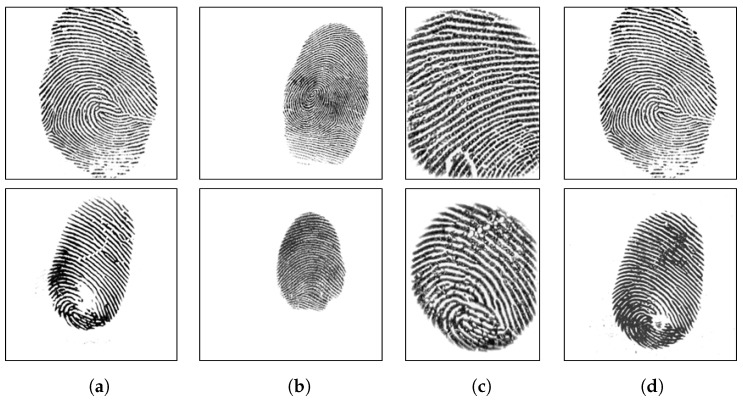
Sample images of LivDet 2015. The top row includes live samples; the bottom row includes fake samples. Samples are from Biometrika (**a**), Crossmatch (**b**), Digital Persona (**c**) and Green Bit (**d**) devices.

LivDet 2015 contains images from Biometrika, Digital Persona, Green Bit and Crossmatch sensor. The models of Biometrika and Digital Persona devices are different from the ones used in 2011 and 2013. The test sets of LivDet 2015 included spoof images of unknown material, i.e., materials that are not included in the training set. Similar to LivDet 2011, all fake fingerprints were made with the cooperative methods. The cast materials were EcoFlex, gelatine, latex, wood glue, liquid EcoFlex, RTV (a two-component silicone rubber), body double, PlayDoh and OOMOO (a silicone rubber). Furthermore, LivDet 2015 has fingerprint images with various resolutions. For fair comparisons between the proposed algorithm and other existing works, we used the original training and test set provided by LivDet. Summaries of these datasets are described in Table 2 and Table 3. Several sample images are shown in Figure 6, Figure 7 and Figure 8.

### 4.2. Experimental Results

In our experiments, three networks were trained, including fPADnet, fPADnet with data augmentation and the original SqueezeNet. All three networks were trained using the following parameters: the learning rate was initialized to 0.0005; the batch size was eight; and the number of epochs was 80. Ten percent of the training samples were used for validation. If the accuracy in the validation set did not increase after four continuous epochs, the learning rate was reduced by half. SqueezeNet and fPADnet were trained on the original training sets provided by the LivDet competitions. As mentioned in [34], image augmentation might boost the performance of the deep networks. In this work, we trained the proposed network with augmented data; this network was called fPADnet with data augmentation. Due to the characteristic of the fingerprint images, only horizontal flip was used for data augmentation. All training and inferencing were implemented using Keras and Tensorflow 1.7. The hardware used to train the network was a desktop with Windows 10, Intel Core i5 3.30 GHz, 12 GB RAM and NVIDIA GTX 1080. Testing was done on a laptop with Windows 10, Intel Core i5 2.30 GHz, 4 GB RAM, to be reasonably comparable to the existing methods. Table 4 summarizes the training pipelines.

**Figure 9:**
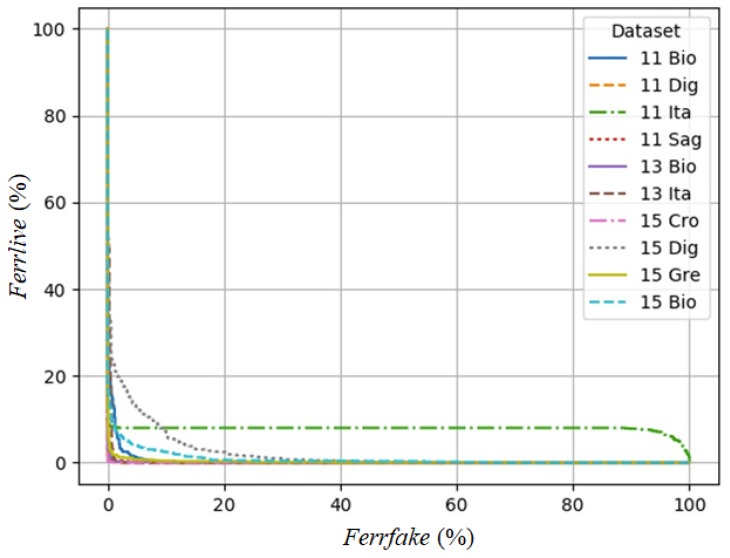
Detection Error Trade-off (DET) graph of fPADnet with error range from zero to 100%.

**Figure 10:**
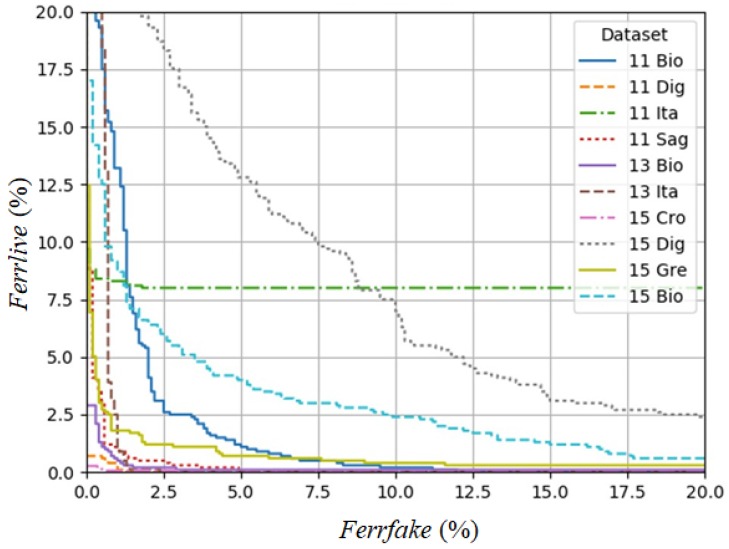
Magnification of DET graph in Figure 9 (focusing on the area of a zero to 20% error rate).

The Fingerprint Liveness Detection Competition uses an Average Classification Error (ACE) to evaluate the performance of participant algorithms. This measurement was used in many existing works. For a fair comparison, the proposed network was assessed using ACE (Equation (2)).

(2) $$ACE=\frac{FerrLive+FerrFake}{2}$$

where *FerrLive* is the rate of misclassified live fingerprints and *FerrFake* is the rate of misclassified fake fingerprints. Figure 9 contains the Detection Error Trade-off (DET) graph, plotting the rate of misclassified live fingerprints (*FerrLive*) vs. the rate of misclassified fake fingerprints (*FerrFake*), of different datasets. Figure 10 is a magnification of Figure 9, which focuses on the area of zero to 20% error rate.

The ACEs of fPADnet on LivDet datasets were compared to the existing works. Table 5 and Table 6 present the comparisons between fPADnet and the most recent algorithms, including non-deep Learning-Based Methods (LBP [25] and local descriptor-based algorithm [23]) and deep learning-based methods (VGG-19 [25]). This comparison shows an improvement of 1.3% in the detection error rate when comparing fPADnet with the state-of-the-art, which is based on VGGNet. Table 5 also proves the effectiveness of Gram-*K* modules. By introducing Gram-*K* modules, fPADnet is about 2.4-times smaller than SqueezeNet, while gaining a lower detection error rate. The proposed network has a small size (around 1.2 MB); thus, it can be stored in low specification systems, such as fingerprint sensors and mobile devices. The average processing time of fPADnet on Nvidia GPU 1080 was 21 ms, which is suitable for real-time systems. The results in Table 6 show that our proposed method could significantly improve the performance in terms of ACE and, especially, the network size, while the runtime was comparable to the others. In Table 6, the ACE of fPADnet and VGG-19 was calculated on three datasets (LivDet 2011, 2013 and 2015); meanwhile, the ACE of AlexNet and LBP was computed on two datasets (LivDet 2011 and 2013). The authors of [25] provided the average processing time of VGG-19, AlexNet and LBP on a single-core machine of 1.8 GHz with 4 GB memory. For a fair comparison, we used a laptop with 2.3 GHz and 4 GB RAM to test our proposed algorithm.

**Figure 11:**
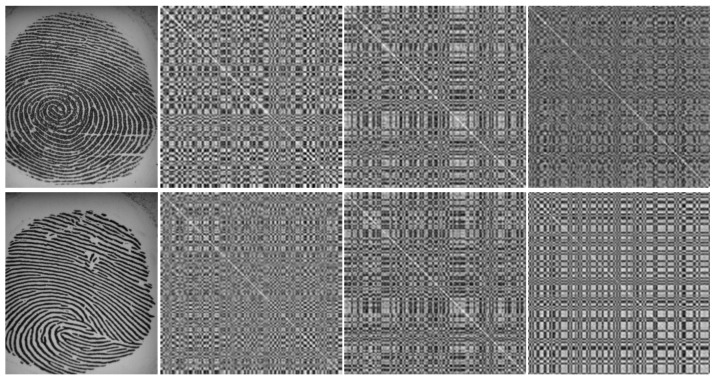
Visualization of Gram matrices (the first row is a live fingerprint, and the second second row is a fake fingerprint).

**Figure 12:**
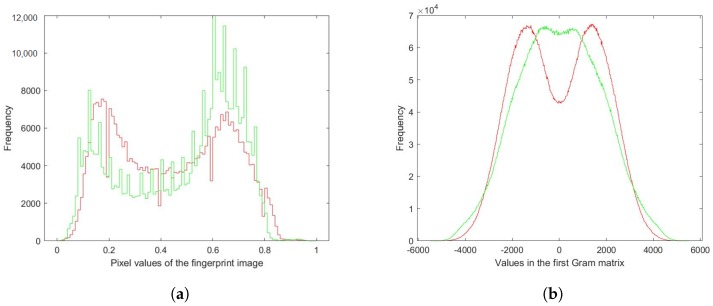

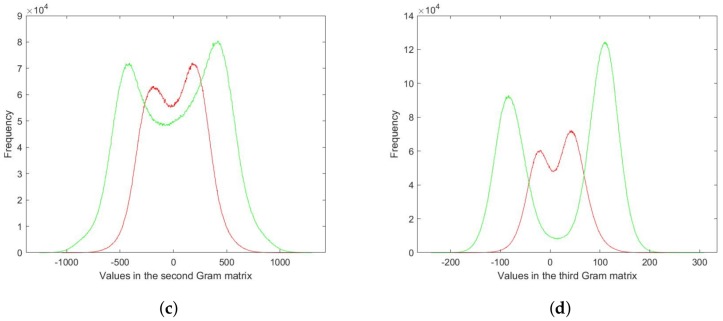
Histogram of fingerprint images (**a**) and three Gram matrices ((**b**)–(**d**)) (red: live, green: fake).

**Figure 13:**
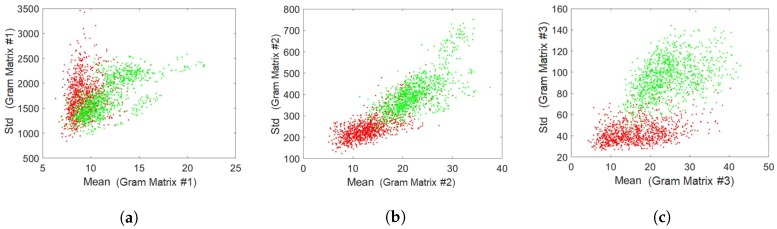
The distribution of mean and standard deviation of: (**a**) The first Gram matrix; (**b**) The second Gram matrix, (**c**) The third Gram matrix (red: live, green: fake).

Figure 11, Figure 12 and Figure 13 are provided with the aim to prove how the Gram matrices were effective for the fingerprint liveness detection. Figure 11 displays a visualization of three Gram matrices in which the top row shows a live fingerprint image (first column) and its Gram matrices: Gram Matrix #1 (second column), Gram Matrix #2 (third column) and Gram Matrix #3 (the last column). The bottom row correspondingly shows the example from a fake image. A collection of 1000 live images and 1000 fake images was randomly picked from the LivDet dataset to build the dataset shown in the two figures. Figure 12 shows the distribution of pixel intensities in the entire collection from the original images and the corresponding Gram matrices in which the red line represents the live and the green line depicts the fake. As seen in Figure 12a, the distribution of pixel intensities of the live and fake is mixed up, meaning that the separation between them is negligible. However, as seen in Figure 12b–d, the Gram matrices deform the distribution to make them more separable. This characteristic is again proven in Figure 13, in which the mean and standard deviation of each sample is plotted. Each sample (an image) is represented by the mean intensity and standard deviation of the pixel intensities. In this sense, the Gram matrix can be considered as a mapping function (such as support vector machines) to project the original data space to another space in which the data are better separable for classification.

## 5. Conclusions

This paper proposed a deep learning-based algorithm to solve the problem of fake fingerprint detection. Since texture has been proven to be one of the most appropriate features to discriminate fake fingerprints, this paper introduced a new module, named Gram-*K*, which is integrated with a deep neural network to extract textural information of fingerprint images. Gram-*K* modules not only extract good features for fake detection, but also help the proposed network be independent of input sizes, i.e., the proposed network can accept images of any size as its inputs. The idea of the Fire module of SqueezeNet was utilized to make the proposed network suitable for low specification fingerprint recognition systems. The experiments proved that the proposed network, fPADnet, is comparable to the state-of-the-art detection accuracy. Besides, it is easier to integrate fPADnet with fingerprint recognition-based applications thanks to its compactness and independence of fingerprint image sizes. Inspired by the success of fPADnet on fake fingerprint detection, we are going to extend fPADnet to other liveness detections, such as face and iris. We expect that there will be a general deep neural network architecture for all the problems mentioned above.

## Figures and Tables

**Table 1:** Structure of the proposed network.

Layer Name/Type	Output Size	Filter Size/Stride (If Not a Fire Layer)	Depth	$s_{1\times 1}$	$e_{1\times 1}$	$e_{3\times 3}$	Number of Parameters
input image	H×W×1						
conv1	$\frac{H}{2}\times \frac{W}{2}\times 96$	7×7/2	1				4800
maxpool1	$\frac{H}{4}\times \frac{W}{4}\times 96$	3×3/2	0				
gram1	128×128×1	1×1	1				12,416
fire2	$\frac{H}{4}\times \frac{W}{4}\times 128$		2	16	64	64	11,920
maxpool2	$\frac{H}{8}\times \frac{W}{8}\times 128$	3×3/2	0				
gram2	128×128×1	1×1	1				16,512
fire3	$\frac{H}{8}\times \frac{W}{8}\times 256$		2	32	128	128	45,344
maxpool3	$\frac{H}{16}\times \frac{W}{16}\times 256$	3×3/2	0				
fire4	$\frac{H}{8}\times \frac{W}{8}\times 384$		2	48	192	192	104,880
gram3	128×128×1	1×1	1				49,280
concatenation	128×128×3		0				
fire5	128×128×128		2	16	64	64	10,432
maxpool5	63×63×128	3×3/2	0				
fire6	31×31×256		2	32	128	128	45,344
maxpool6	31×31×256	3×3/2	0				
conv7	31×31×2	1×1/1	1				
avgpool7	1×1×2	31×31/1	0				
Total number of parameters							300,928
Total number of parameters if batch normalization is applied							308,554

**Table 2:** Summary of the datasets used in the experiments (1).

Dataset	Sensor	Model	Image Size
LivDet 2011	Biometrika	FX2000	315×372
	ItalData	ET10	640×480
	Digital Persona	4000B	355×391
	Sagem	MSO300	352×384
LivDet 2013	Biometrika	FX2000	315×372
	ItalData	ET10	640×480
LivDet 2015	Green Bit	DactyScan26	500×500
	Biometrika *	HiScan-PRO	1000×1000
	Digital Persona	U.are.U 5160	252×324
	Crossmatch	L Scan Guardian	640×480

* All images are 500 dpi, except for the ones in LivDet 2015 Biometrika, which are 1000 dpi.

**Table 3:** Summary of the datasets used in the experiments (2).

Dataset	Sensor	Training Samples (Live/Spoof)	Test Samples (Live/Spoof)	Cast Materials
LivDet 2011	Biometrika	1000/1000	1000/1000	Gelatin, latex, EcoFlex, silicon, wood glue
	ItalData	1000/1000	1000/1000	
	Digital Persona	1000/1000	1000/1000	Gelatin, latex, PlayDoh, silicon, wood glue
	Sagem	1000/1000	1000/1000	
LivDet 2013	Biometrika	1000/1000	1000/1000	Gelatin, latex, EcoFlex, modasil, wood glue
	ItalData	1000/1000	1000/1000	
LivDet 2015 *	Green Bit	1000/1000	1000/1500	EcoFlex, gelatine, latex, wood glue, liquid EcoFlex, RTV
	Biometrika	1000/1000	1000/1500	
	Digital Persona	1000/1000	1000/1500	
	Crossmatch	1500/851	1500/1448	Body double, EcoFlex, PlayDoh, OOMOO, gelatine

* The LivDet 2015 Green Bit, Biometrika and Digital Persona training sets do not include fake fingers from Liquid EcoFlex and RTV. * The LivDet 2015 Crossmatch training set does not include fake fingers from OOMOO and gelatine.

**Table 4:** Summary of training parameters.

Training Pipeline	Training Sets	Hyperparameters for Training
SqueezeNet	LivDet	Learning rate = 0.0005 * Batch size = 8 Number of epochs = 80 Validation data = 10% of training data
fPADnet	LivDet	
fPADnet with data augmentation	LivDet & augmented images	

* The learning rate is reduced by half if the validation error does not decrease after four continuous epochs.

**Table 5:** Performance comparison on Average Classification Error (ACE). LBP, Local Binary Pattern.

Dataset	Sensor	Average Classification Errors (%)					
		LBP	Local Descriptor	VGG-19	SqueezeNet	fPADnet	fPADnet (with Augmentation)
LivDet 2011	Biometrika	8.8 [25]	6.8 [23]	8.0 [25]	4.7	2.8	5.0
	ItalData	12.3 [25]	13.7 [23]	3.2 [25]	6.4	5.0	4.8
	Digital Persona	4.1 [25]	3.6 [23]	5.2 [25]	3.1	0.6	2.0
	Sagem	7.5 [25]	4.9 [23]	1.7 [25]	2.3	1.5	2.6
	Average	8.2	7.3	4.5	4.1	2.5	3.6
LivDet 2013	Biometrika	1.7 [25]	0.6 [23]	1.8 [25]	1.2	0.9	0.7
	ItalData	2.3 [25]	0.6 [23]	0.4 [25]	1.3	1.3	0.9
	Average	2.0	0.6	1.1	1.3	1.1	0.8
LivDet 2015	Green Bit	-	-	4.6 [10]	4.9	1.4	2.5
	Biometrika	-	-	5.6 [10]	1.9	4.1	3.8
	Digital Persona	-	-	6.3 [10]	2.9	8.5	7.0
	Crossmatch	-	-	1.9 [10]	5.9	0.3	3.4
	Average	-	-	4.6	3.9	3.6	4.2
Overall Average		-	-	3.9	3.5	2.6	3.3

The best performance for each dataset (each row) is underlined.

**Table 6:** Comparison of network size and processing time.

Algorithm	ACE ${}^{1}$	Number of Parameters in the Network (Million)	Processing Time (ms)		CPU Specification
			GPU ${}^{2}$	CPU	
fPADnet	2.6	0.5	21	241	2.3 GHz, 4 GB RAM
VGG-19 [25]	3.9	140	-	650	1.8 GHz, 4 GB RAM
AlexNet [25]	4.1	60	-	230	
LBP [25]	6.1	-	-	50	

${}^{1}$ The ACE rates of AlexNet and LBP were computed on LivDet2011 and LivDet2013. The ACE rates of fPADnet and VGG-19 were computed on LivDet2011, LivDet2013 and LivDet2015. ${}^{2}$ NVIDIA GTX 1080.

## Abbreviations

The following abbreviations are used in this manuscript:

CNN	Convolutional Neural Network
FPRS	Fingerprint Recognition System
LivDet	International Fingerprint Liveness Detection competition
